# Dietary Supplementation with Curcumin Reduce Circulating Levels of Glycogen Synthase Kinase-3β and Islet Amyloid Polypeptide in Adults with High Risk of Type 2 Diabetes and Alzheimer’s Disease

**DOI:** 10.3390/nu12041032

**Published:** 2020-04-09

**Authors:** Rohith N Thota, Jessica I Rosato, Cintia B Dias, Tracy L Burrows, Ralph N Martins, Manohar L Garg

**Affiliations:** 1 Nutraceuticals Research Program, School of Biomedical Sciences & Pharmacy, University of Newcastle, Callaghan, NSW 2308, Australia; rohith.thota@newcastle.edu.au (R.N.T.); jessica.rosato@uon.edu.au (J.I.R.); Cintia.botelhodias@mq.edu.au (C.B.D.); tracy.burrows@newcastle.edu.au (T.L.B.); 2 Riddet Institute, Massey University, Palmerston North 4474, New Zealand; 3 School of Health Sciences, University of Newcastle, Callaghan, NSW 2308, Australia; 4 School of Biomedical Sciences, Macquarie University, Macquarie, NSW 2109, Australia; ralph.martins@mq.edu.au

**Keywords:** curcumin, glycogen synthase kinase-3, insulin resistance, Islet amyloid polypeptide, type 2 diabetes mellitus

## Abstract

Dietary supplementation with curcumin has been previously reported to have beneficial effects in people with insulin resistance, type 2 diabetes (T2D) and Alzheimer’s disease (AD). This study investigated the effects of dietary supplementation with curcumin on key peptides implicated in insulin resistance in individuals with high risk of developing T2D. Plasma samples from participants recruited for a randomised controlled trial with curcumin (180 mg/day) for 12 weeks were analysed for circulating glycogen synthase kinase-3 β (GSK-3β) and islet amyloid polypeptide (IAPP). Outcome measures were determined using ELISA kits. The homeostasis model for assessment of insulin resistance (HOMA-IR) was measured as parameters of glycaemic control. Curcumin supplementation significantly reduced circulating GSK-3β (−2.4 ± 0.4 ng/mL vs. −0.3 ± 0.6, *p* = 0.0068) and IAPP (−2.0 ± 0.7 ng/mL vs. 0.4 ± 0.6, *p* = 0.0163) levels compared with the placebo group. Curcumin supplementation significantly reduced insulin resistance (−0.3 ± 0.1 vs. 0.01 ± 0.05, *p* = 0.0142) compared with placebo group. Dietary supplementation with curcumin reduced circulating levels of IAPP and GSK-3β, thus suggesting a novel mechanism through which curcumin could potentially be used for alleviating insulin resistance related markers for reducing the risk of T2D and AD.

## 1. Introduction

Insulin resistance is of particular interest as defective insulin signalling in the brain contributes to the accumulation of amyloid beta (Aβ) and tau protein, presenting a pathophysiological link between Alzheimer’s disease (AD) and type 2 diabetes (T2D) [1]. It has been previously described as diabetes of the brain, or type 3 diabetes [2]. The prevalence of insulin resistance is growing exponentially, with over 463 million people suffering from diabetes worldwide according to the recent edition of the International Diabetes Federation Atlas 2019, of which >90% are T2D. Recent evidence from a population-based study shows a link between T2D and AD, with an incidence 2–5 times higher in those with T2D [3] and hyperinsulinemia [4].

Islet amyloid polypeptide (IAPP) is a peptide hormone co-secreted with insulin by pancreatic β-cells [5]. Elevated serum levels of IAPP is a pathological hallmark of insulin resistance and correlates with AD diagnosis [6]. Furthermore, the amyloidosis of IAPP involving formation of Aβ-like structures and extracellular deposits of amyloid in the pancreas is a distinctive feature of T2D [5]. IAPP can also induce peripheral insulin resistance by antagonising insulin activity, further linking to the overexpression of glycogen synthase kinase-3 (GSK-3) [7]. Impaired insulin signalling and subsequent hyperactivity of GSK-3 in rodent and human models have been associated with the accumulation of Aβ and tau protein in the brain [8]. Recent research has uncovered a pathophysiological link between T2D and AD involving insulin resistance and the activation of GSK-3, a serine-threonine kinase involved in a multitude of physiological processes including glycogen metabolism and microtubule stability [9,10]. GSK-3 produces two isoforms (α and β) upon activation. Along with its pleiotropic roles in human physiology in skeletal muscle and liver, GSK-3 is linked to cognitive disorders and is thought to play an important role in the pathogenesis of AD [10]. Insulin triggers the phosphorylation (inactivation) of GSK-3 via the PI3k/Akt signalling cascade, while defective insulin signalling results in decreased phosphorylation and consequently elevated activation of GSK-3 in the brain [11]. Over-activity of GSK-3α mainly enhances plaque-associated aggregation of insoluble Aβ, while GSK-3β primarily contributes to the hyper-phosphorylation of tau [11]. The purported role of GSK-3 in the development of AD has led to it being investigated as a potential therapeutic target.

Curcumin is a bio-active curcuminoid, extracted from the rhizomes of turmeric with a wide range of pharmacological properties including the ability to reduce inflammation, oxidative stress and insulin resistance [12,13,14]. In vivo studies in animal models [13] and a few clinical trials [15] have shown beneficial effects of curcumin on insulin resistance. Systematic reviews have provided strong evidence for investigating curcumin efficacy for management of type 2 diabetes mellitus [15]. Substantial in vitro data are available on the anti-oxidant anti-inflammatory activities of curcumin, suggesting a possible link to its protective effect on dementia and AD [16]. In animal models, curcumin has been shown to reduce systemic inflammatory markers (cyclooxygenase (COX-2) and phospholipases; transcription factors such as nuclear factor kappa-B; pro-inflammatory cytokines such as tumour necrosis factor-α and IL-1β and C-reactive protein (CRP) concentrations) suggesting a possible link between its anti-inflammatory and cognitive protection effects [17]. Curcumin has been proven to have strong antioxidant action by the inhibition of the formation and of free radicals [18]. It decreases the oxidation of low-density lipoprotein that cause the deterioration of neurons, not only in AD but also in other neuron degenerative disorders (Huntington’s and Parkinson’s disease) [18]. Curcumin also increased memory function and non-spatial memory related parameters in aged rodent models with cognitive impairments [19,20,21]. Curcumin inhibits the activity of activator protein-1, a transcription factor implicated in the expression of Aβ [22]. Increasing evidence suggests that curcumin supplementation mitigates Aβ deposition and tauopathy whilst exerting inhibitory effects on GSK-3 activity via interactions with the PI3k/Akt cascade [23]. Epidemiologic studies also suggest a link between curcumin and cognitive benefits. Ng et al. [24] observed that subjects with higher curry (curcumin is a common ingredient) consumption had 6% higher Mini-Mental State Examination (MMSE) scores compared with subjects who never or rarely consume curry.

The widespread use of curcumin as an additive, the relatively high safety profile established in a number of short-term trials and the potency of curcumin to suppress insulin resistance could be a beneficial factor in management of both T2D and AD. The aim of this study was to determine if dietary supplementation with curcumin reduce plasma levels of peptides, GSK-3β and IAPP that are implicated in the insulin resistance in people at a high risk of developing T2D.

## 2. Materials and Methods

### 2.1. Participants

Participants were recruited for the purposes of the curcumin and omega-3 fatty acids for prevention of type 2 diabetes (COP-D) study [25] from the Hunter region in New South Wales, Australia. Interested participants were screened through telephone interviews per the inclusion and exclusion criteria. If eligible, potential participants were posted self-administered health/medical, diet, and physical activity questionnaires as well as a consent form. Inclusion criteria for the current study included: aged of 30–70 (years); body mass index (BMI) of 25–45 kg/m^2^; ≥12 score in the Australian Type 2 Diabetes Risk (AUSDRISK) questionnaire (a non-invasive questionnaire for assessing the risk of developing type 2 diabetes); diagnosed with either impaired fasting glucose (IFG, fasting glucose of 6.1–6.9 mmol/L) or impaired glucose tolerance (IGT, 2-h plasma glucose ≥7.8 mmol/L and <11.1 mmol/L) or both; and glycosylated haemoglobin (HbA1c) levels of 5.7–6.4%. Participants were excluded if they were unwilling to provide blood samples at the baseline and post-intervention (12-week) site visits; diagnosed with T2D; gallbladder problems; pacemaker implants; severe neurological diseases or seizures; pregnant, planning to become pregnant or breastfeeding/lactating; taking any dietary supplements (such as fish oil, cinnamon, probiotics, vitamin D, chromium, etc.) known to influence blood glucose levels; consuming ≥2 servings of oily fish per week; or taking any medications known to have drug-nutrient interactions with curcumin (blood thinning medications such as Aspirin and warfarin). All participants gave their written informed consent. The study was conducted in accordance with the Declaration of Helsinki, and has been approved by the University of Newcastle Human Research Ethics Committee (H-2014-0385). The trial is registered with the Australia & New Zealand Clinical Trial Registry (ACTRN12615000559516).

### 2.2. Study Design

The detailed protocol of the current study is previously published [25]. Screened participants were randomised to placebo (2 × placebo tablets matching for curcumin) and curcumin (2 × 500 mg curcumin tablets [Meriva^®^] providing 180 mg of curcumin per day). Participant compliance was measured during the follow-up (6-week) and post-intervention (12-week) site visits via a capsule count-back method and capsule intake log. Any illnesses, changes in medications or medical diagnoses during the study timeframe were recorded.

### 2.3. Data Collection and Outcome Measures

The primary outcome of this study was to evaluate the effects of curcumin on circulating levels of GSK-3β in adults at a high risk of developing T2D. Fasting (≥10-h) blood samples were collected from participants at baseline and post-intervention (12-week), at either the Nutraceuticals Research Program clinical trial facility or John Hunter Hospital in Newcastle, NSW. These samples were analysed using GSK-3β enzyme linked immunosorbent assay (ELISA) kits, which have high specificity for human GSK-3β and no detectable cross-reactivity with other relevant proteins (manufacturer, Aviva systems biology; detection range, 0.625–40 ng/mL; mean intra-Assay CV, <10%; and mean inter-Assay CV, <12%). The secondary outcome was to evaluate the effects of curcumin supplements on IAPP, which was analysed via ELISA (manufacturer, Aviva systems biology; detection range, 0.156–10 ng/mL; mean intra-assay CV%, <4.6%; Mean inter-assay CV%, <7.4%). Serum insulin and glucose were measured by Hunter Area Pathology Services using radio immunoassay technique. HOMA2-IR was calculated using the Diabetes Trials Unit online calculator.

#### Questionnaires (Diet, Physical Activity and Medical History)

Medical history data collected include medication and supplement use as well as family medical history. Demographic characteristics collected from participants include age, sex and ethnicity. Participants were advised to maintain their regular dietary pattern and physical activity levels throughout the 12-week study period. To estimate their habitual dietary patterns, participants were asked to complete a 3-day (2 weekdays plus 1 weekend day) food diary prior to all three site visits. Food diaries were analysed using FoodWorks Xyris (version 8.0) to assess changes in energy intakes (kJ) during the study period. Habitual physical activity (METs, minutes/week) was assessed using the International Physical Activity Questionnaire (IPAQ) long form version (2002), which was completed by participants prior to all three site visits.

### 2.4. Body Composition and Anthropometric Measures

Body composition measurements included body weight (kg), muscle mass (kg), body fat mass (kg), body mass index (BMI, kg/m^2^) and body fat per cent (%). Body composition was measured using direct segmental multi-frequency bioelectrical impedance (InBody 230, Biospace Co., Ltd. Seoul, Korea). Anthropometric measurements included height (cm; SE206, Seca), BMI (kg/m^2^) and waist-circumference (cm).

### 2.5. Statistical Analysis

For the purposes of the current sub-study, n = 29 (15 allocated to placebo group and 14 allocated to curcumin treatment group) participants were analysed for GSK-3β and IAPP resulting in a study power of 97.7% for the detection of a 2.4 ng/mL reduction in serum GSK-3β level (1.3 ng/mL SD, *p* value = 0.01). Data collected at baseline were analysed for normality using histograms with a normal distribution curve overlaid the Shapiro–Wilk test, and are presented as mean ± SEM (standard error of the mean) or median (IQR, interquartile range) as appropriate. Significant changes in the baseline data between the two intervention groups were assessed through t-test or Mann–Whitney U Test when the normality assumption was not met. Post-intervention data are presented as mean ± SEM or median (IQR) of absolute change (post-intervention value minus baseline value). Changes from baseline to post-intervention within treatment groups were assessed through t-test or Wilcoxon signed-rank test.

## 3. Results

### 3.1. Baseline Characteristics

Twenty-nine serum samples were analysed from the COP-D trial for the primary outcome, GSK-3β, and for IAPP. No significant differences were observed between the two participant groups for all baseline characteristics, including demographics and serum outcome measures (Table 1). All participants were insulin resistant, with an average fasting glucose of 5.4 ± 0.1 mmol/L and median fasting serum insulin of 9.9 (4.9) mIU/L. Trial participants had a median GSK-3β of 3.0 (1.7) ng/mL and median IAPP of 4.5 (2.6) ng/mL. Likewise, there were no significant changes in dietary intake or physical activity within and between the two groups post-intervention (Table 2). Comparisons between the placebo and curcumin group showed no significant differences in the body composition and anthropometric measurements collected at baseline and post intervention (Table 1 and Table 2). The average BMI of participants at baseline was within the obese category (≥30 kg/m^2^) and was accompanied by high body fat per cent (34.7 ± 1.8%) and waist circumference (105.4 ± 2.4 cm). There were no significant changes in body composition and anthropometric measurements post-intervention (Table 2). According to the capsule count, mean compliance to the randomised intervention was 94.9 ± 5.80%. Curcumin was well tolerated by participants and no adverse events due to the allocated intervention were reported during the 12-week study period.

### 3.2. Effects of Curcumin on GSK-3β and IAPP

**Figure 1:**
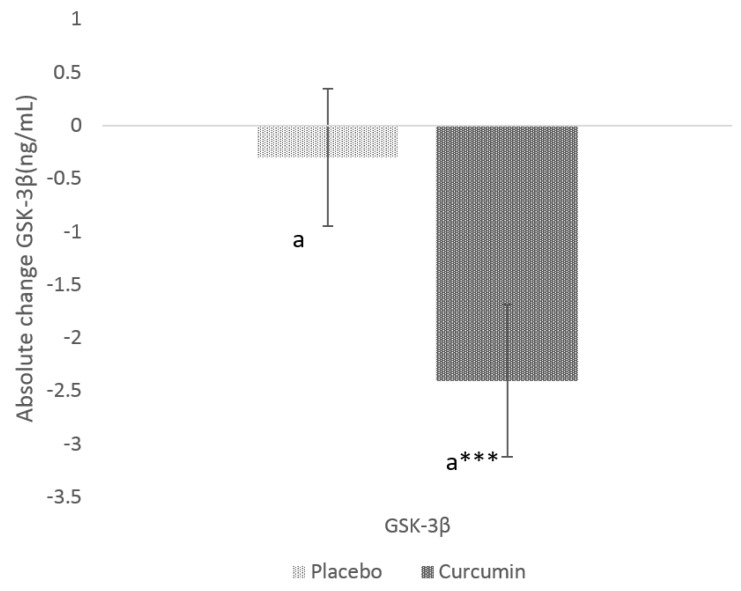
Absolute change in serum glycogen synthase kinase –β (GSK-3β) from baseline to post-intervention in placebo and curcumin groups for 12 weeks. *** *p* < 0.001 represents the difference within the treatment group. Small letter (a) represents the significant difference between the treatment groups.

**Figure 2:**
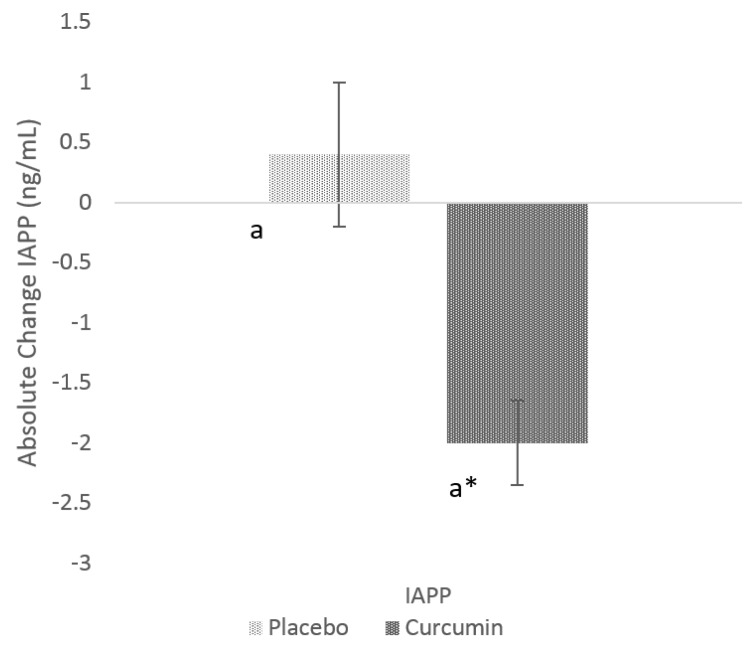
Absolute change in serum islet amyloid peptide (IAPP) from baseline to post-intervention in placebo and curcumin groups for 12 weeks. * *p* < 0.05 represents the difference within the treatment group. Small letter (a) represents the significant difference between the treatment groups.

After 12- weeks of curcumin supplementation, circulating GSK-3β levels were significantly lower in the curcumin group (−2.4 ± 0.4 ng/mL, *p* value = 0.00001) (Figure 1). When compared to placebo, there was also significant (*p* value = 0.0068) reduction in serum GSK-3β levels in the curcumin group. Similar observation was found with IAPP (−2.0 ± 0.7 ng/mL, *p* value 0.01) within curcumin group post-intervention (Figure 2); When compared to PL, there was also a significant (*p* value = 0.0163) change in mean IAPP.

### 3.3. Glycaemic Indices

**Figure 3:**
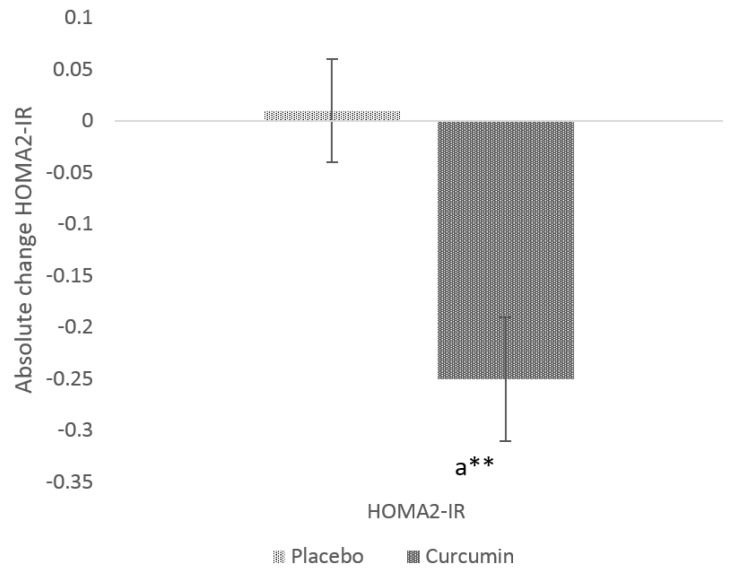
Absolute change in HOMA2-IR from baseline to post-intervention in placebo and curcumin groups for 12 weeks. ** *p* < 0.01 represents the difference within the treatment group. Small letter (a) represents the significant difference between the treatment groups.

Serum insulin was significantly reduced (*p* value = 0.0076) in the curcumin treatment group (−1.9 µIU) from baseline (Table 2) and was also significantly different from placebo (0.0115). Similar trends were observed with respect to HOMA2-IR (Figure 3) in curcumin group. Post-intervention, significant changes (*p* value = 0.0142) in HOMA2-IR were only observed in the curcumin treated group (−0.11 ± 0.05) compared to the placebo group. No significant changes were observed on blood glucose levels after supplementation with curcumin (*p* = 0.9747) compared to the placebo group.

## 4. Discussion

**Figure 4:**
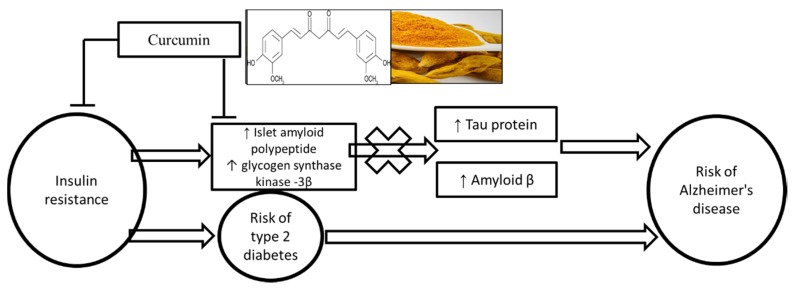
Summary of potential mechanism of curcumin in reducing the risk of type 2 diabetes and Alzheimer’s disease.

The primary finding of this study is that oral supplementation with curcumin (180 mg per day) for 12 weeks reduces the circulating levels of peptides that are implicated in insulin resistance, namelt GSK-3β and IAPP (Figure 4). In addition, we demonstrated that curcumin supplementation positively affects glycaemic control via reduction in insulin resistance and fasting serum insulin compared with the placebo group.

The aetiology of insulin resistance is dependent on multiple of factors [26] and there is substantial evidence implicating the role of GSK-3 in insulin resistance [27]. In T2D, GSK-3 is an enzyme of glycogen synthesis, which plays a key role in regulating blood glucose. Its role in insulin deficiency and insulin resistance [28] is implicated via insulin/PI3-kinase/protein kinase B (insulin/PI3K/Akt) signalling pathway [29]. In AD, GSK-3β is involved in the hyperphosphorylation of microtubule-associated protein tau (tau), which is one of the pathological features in AD [30].

There are multiple potential reasons for evaluating the effects of curcumin on serum GSK-3β levels. Pre-clinical studies indicated that curcumin supplementation reduced GSK-3 activity resulting in protection against Aβ accumulation and hyper-phosphorylation of tau [23,31,32]. Simulated docking studies have shown optimal binding capacity of curcumin with GSK-3β and interactions with key amino acids resulting in the deactivation of the kinase [33]. Glycogen metabolism is a highly regulated process in which GSK-3β plays an essential role [33]. Follow-up in vitro and in vivo studies have confirmed curcumin’s pharmacological activities by demonstrating that this bioactive potently inhibits GSK-3β (IC_50_ = 66.3 nM) and increases fasting liver glycogen levels [33]. A recent randomised placebo-controlled trial with curcumin (90 mg), similar to the current study dose, demonstrated reduction in Aβand tau in a certain brain areas leading to the improvement memory and attention in adults aged 51–84 years [33]. Amyloid and tau accumulation on brain were assessed by positron emission tomography (FDDNP-PET). Curcumin significantly lowered binding in the amygdala (ES =  −0.41, *p*  =  0.04) compared with a placebo [34]. In line with these observations from the pre-clinical reports, in the current study we showed that dietary supplementation with curcumin significantly reduce serum levels of GSK-3β in adults with insulin resistance. Follow-up studies are required to determine if this effect has direct implications in reducing the risk for T2D and AD.

IAPP, a pancreatic beta cell peptide, can evoke insulin resistance by antagonising insulin in a non-competitive manner [7,35]. Although IAPP has been previously shown to have no significant effect on glucose transport, it decreased insulin-stimulated glucose transport by about 30% [35]. IAPP also increased GSK-3 activity, which in turn led to increased phosphorylation of glycogen synthase and decreased glycogen synthesis de novo [7]. IAPP mediates several important brain functions via binding to its receptor in the brain, including regulating glucose metabolism, inflammatory responses, and potentially in neurogenesis [36,37]. However, amylin can aggregate when concentrations are high and become neurotoxic in cell cultures and is associated with brain amyloid burden and cognitive impairment in AD [38,39]. Recent studies have also indicated that the high plasma levels of IAPP is a pathological hallmark of insulin resistance [40] and correlates with AD diagnosis and brain structure [41]. In vitro studies revealed that curcumin significantly reduces h-IAPP fibril formation and aggregates formed in the presence of curcumin display alternative structure compared to the actual peptide [42]. Curcumin increased the time required for the conversion of IAPP monomer to assemblies that are visible in NMR, including β-sheet, suggesting that curcumin has the potential to inhibit the formation of the oligomers that are on-pathway to formation of amyloid [42]. In the current study, we reported if curcumin has a direct effect on the plasma levels of IAPP. Curcumin supplementation significantly reduced the plasma IAPP levels in the current study, providing further insights into the beneficial effects of curcumin on glycaemic homeostasis.

Insulin resistance and hyperinsulinemia is associated with the development of both T2D and AD [43]. Converging evidence from cross-sectional studies has shown significant associations of HOMA-IR with mild cognitive impairment and AD [44,45]. Moreover, presence of IR accelerates the formation of Neuritic plaques which are involved in the pathogenic process of AD [45]. Curcumin has repeatedly demonstrated efficacy in regard to improving insulin resistance [15]. Curcumin treatment reduced both serum insulin and insulin resistance (measured via HOMA2-IR). A similar effect of curcumin on insulin resistance was observed in a nine-month randomised controlled trial with curcumin extract in a pre-diabetic population [46]. Curcumin intervention reduced insulin resistance, as indicated by an increased HOMA-IR and reduced C-peptide levels [47]. Another three-month randomised controlled trial with overweight/obese T2DM patients has also indicated that the curcumin supplementation group resulted in a significant reduction of fasting glycaemia and insulin resistance [48]. However, in contrast to these, we did not observe any significant reductions in fasting glucose with curcumin supplementation. Curcumin mediated reduction in the insulin levels and insulin resistance (HOMA-IR) could reduce the risk factors for cognitive impairment such as neuritic plaques and amyloid formation. As IR and hyperinsulinemia are significantly associated and there is a common molecular pathway for T2D and AD, this study’s findings on the effect of curcumin on insulin resistance implicates a potential role of curcumin in reducing the risk for AD. In this study, we provided novel insights on the efficacy of curcumin in insulin resistance by providing evidence on regulation of key peptides such as GSK-3β and IAPP, which play an important role in insulin resistance.

Participants compliance to the intervention in the current study was high. Aviva Systems Biology GSK-3β ELISA kits used to measure the primary outcome have high specificity for human GSK-3β and no detectable cross-reactivity with other relevant proteins. The link between AD and T2D is a relatively new and emerging area of research. In this study, we were able to provide novel potential adjuvants for ameliorating common risk factors underlying T2D and AD. However, the results of the current sub-study are limited by their preliminary nature, and as such a follow-up study is required to substantiate effects of curcumin supplementation on GSK-3β and IAPP and whether it can affect neurological/metabolic parameters specific to T2D and AD in high-risk adults. Furthermore, the preliminary results of this sub-study may not be generalisable or transferable to other populations as only adults with insulin resistance and high risk of T2D were studied.

## Figures and Tables

**Table 1:** Baseline characteristics of the trial participants.

Characteristics	Total (n = 29)	Placebo (n = 15)	Curcumin (n = 14)	*p* Value
Age (years)	52.3 ± 1.9	50.4 ± 2.6	54.5 ± 2.9	0.2998
Males/females (n/n)	12/17	6/9	6/8	-
Ethnicity—no (%)				
Caucasian	23	12 (80)	11 (78.6)	-
Asian	3	1 (6.7)	2 (14.3)	-
Others	3	2 (13.3)	1 (7.1)	-
Anthropometry measures				
Body weight (kg)	88.8 ± 3.0	90.7 ± 4.9	86.7 ± 3.5	0.5206
Muscle mass (kg)	33.3 ± 1.4	32.4 ± 1.7	34.4 ± 2.4	0.4998
Body fat mass (kg)	32.4 ± 2.2	33.7 ± 3.5	31.1 ± 3.0	0.5785
Body mass index (kg. m^−2^)	31.3 ± 1.0	32.3 ± 1.7	30.2 ± 1.1	0.3276
Waist circumference(cm)	105.4 ± 2.4	106.0 ± 3.9	104.9 ± 2.9	0.8246
Percent body fat (%)	34.7 ± 1.8	35.3 ± 2.2	34.8 ± 2.5	0.5467
Plasma outcome measures				
Fasting glucose (mmol/L)	5.4 ± 0.1	5.2 ± 0.1	5.6 ± 0.2	0.1121
Fasting serum insulin (mIU/L)	9.9 (4.9)	10.3 (7.9)	9.1 (4.6)	0.6005
HOMA2-IR	1.3 (0.6)	1.3 (1.1)	1.2 (0.6)	0.7268
IAPP (ng/mL)	4.5 (2.6)	4.1 (2.6)	3.9 (3.1)	0.8948
GSK-3β (ng/mL)	3.0 (1.7)	2.7 (1.8)	3.4 (2.7)	0.1625
Dietary intakes (kj)	9047.3 ± 424.9	8497.1 ± 599.6	9682.1 ± 573.06	0.1685
Physical Activity (METs-minutes/week)	2432 (4920)	3894 (5214)	1765 (1597)	0.1161

Data are presented as mean ± SEM or median (IQR) for continuous variables and n (%) for categorical variables. n, number of participants; METs, metabolic equivalents; SEM, standard error of the mean; IAPP, islet amyloid polypeptide; GSK-3β, glycogen synthase kinase-3 beta. *p*-values represent the significant differences between the groups.

**Table 2:** Changes in the study parameters of the participants in placebo and curcumin group after three-month intervention period.

Outcome Measures	Treatment Group	Mean Change	*p* Value	Mean Difference between Treatment Groups	*p* Value
Body weight (kg)	Placebo	0.64 ± 0.4	0.1731		
	Curcumin	−0.1 ± 0.4	0.8272	−0.7 ± 0.6	0.2292
Muscle mass (kg)	Placebo	0.1 (0.1)	0.8902		
	Curcumin	0.25 (0.7)	0.4440	0.1(0.8)	0.5257
Body fat mass (kg)	Placebo	0.1 (0.4)	0.7577		
	Curcumin	−0.85 (0.9)	0.8487	−0.5 (2.1)	0.3478
Body mass index (kg/m^2^)	Placebo	0.20 (0.2)	0.1945		
	Curcumin	0.03 (0.2)	0.8296	0 (0.7)	0.3573
Waist circumference (cm)	Placebo	0.87 ± 0.7	0.2258		
	Curcumin	−0.10 ± 0.8	0.8940	−0.1 ± 1.0	0.3557
Percent body fat (%)	Placebo	0.5 (1.8)	0.4388		
	Curcumin	−0.6 (1.6)	0.5980	0 (2)	0.7699
Fasting glucose (mmol/L)	Placebo	−0.06 ± 0.1	0.3625		
	Curcumin	−0.07 ± 0.1	0.6041	−0.004 ± 0.1	0.9747
Fasting serum insulin (µIU/L)	Placebo	0.1 ± 0.4	0.8251		
	Curcumin	−1.9 ± 0.6	0.0076	−2.0 ± 0.4	0.0115
Dietary intakes (kj)	Placebo	−134.5 ± 479.2	0.7830		
	Curcumin	298.4 ± 487.9	0.5520	−433.0 ± 686.6	0.5338
Physical activity (Metabolic equivalent-minute/week)	Placebo	−473 (3880)	0.2202		
	Curcumin	104.5 (1508)	0.6249	0 (2618)	0.2386

Data are presented as mean ± SEM or median (IQR) as appropriate. *p*-values represent the significant differences with-in and between the groups.

## Abbreviations

AUSDRISK	Australian Type 2 Diabetes Risk
AD	Alzheimer’s disease
GSK-3β	glycogen synthase kinase-3 β
HOMA-IR	homeostasis model for assessment of insulin resistance
IAPP	islet amyloid polypeptide
T2D	type 2 diabetes
